# Sensorimotor Oscillations Prior to Speech Onset Reflect Altered Motor Networks in Adults Who Stutter

**DOI:** 10.3389/fnhum.2016.00443

**Published:** 2016-09-02

**Authors:** Anna-Maria Mersov, Cecilia Jobst, Douglas O. Cheyne, Luc De Nil

**Affiliations:** ^1^Department of Speech-Language Pathology, University of TorontoToronto, ON, Canada; ^2^Program in Neurosciences and Mental Health, Hospital for Sick Children Research InstituteToronto, ON, Canada; ^3^Department of Medical Imaging, University of TorontoToronto, ON, Canada

**Keywords:** magnetoencephalography, developmental stuttering, beta suppression, beta synchronization, speech preparation

## Abstract

Adults who stutter (AWS) have demonstrated atypical coordination of motor and sensory regions during speech production. Yet little is known of the speech-motor network in AWS in the brief time window preceding audible speech onset. The purpose of the current study was to characterize neural oscillations in the speech-motor network during preparation for and execution of overt speech production in AWS using magnetoencephalography (MEG). Twelve AWS and 12 age-matched controls were presented with 220 words, each word embedded in a carrier phrase. Controls were presented with the same word list as their matched AWS participant. Neural oscillatory activity was localized using minimum-variance beamforming during two time periods of interest: speech preparation (prior to speech onset) and speech execution (following speech onset). Compared to controls, AWS showed stronger beta (15–25 Hz) suppression in the speech preparation stage, followed by stronger beta synchronization in the bilateral mouth motor cortex. AWS also recruited the right mouth motor cortex significantly earlier in the speech preparation stage compared to controls. Exaggerated motor preparation is discussed in the context of reduced coordination in the speech-motor network of AWS. It is further proposed that exaggerated beta synchronization may reflect a more strongly inhibited motor system that requires a stronger beta suppression to disengage prior to speech initiation. These novel findings highlight critical differences in the speech-motor network of AWS that occur prior to speech onset and emphasize the need to investigate further the speech-motor assembly in the stuttering population.

## Introduction

Adults who stutter (AWS) have demonstrated atypical activation and structural patterns in regions comprising the speech-motor network (Ludlow and Loucks, 2003; Brown et al., 2005; Ingham et al., 2012; Belyk et al., 2015). For example, AWS have demonstrated reduced activation of the left inferior frontal gyrus (BA 47) and bilateral superior and middle temporal gyri, as well as over-activation of the motor cortex, primarily in the right hemisphere (Ludlow and Loucks, 2003; Brown et al., 2005; Ingham et al., 2012; Belyk et al., 2015). AWS also showed reduced white matter tracts underlying the left ventral premotor cortex, left rolandic operculum and left arcuate fasciculus (Cai et al., 2014b; Belyk et al., 2015; Cieslak et al., 2015; Connolly et al., 2015). The authors have proposed that such patterns result in an atypical coordination of motor and sensory regions required for speech production. However, speech production tasks in AWS have been primarily investigated using hemodynamic imaging methods (e.g., fMRI) with a low temporal resolution of several seconds (Aguirre et al., 1998; Dale et al., 2000), limiting the ability to observe the way in which activation of the speech motor network of AWS unfolds prior to the actual onset of speech production. Characterizing processes associated with the preparation for speech are an important step to understanding that which occurs prior to a dysfluent utterance and is critical to our understanding of fluency disruptions. The current work examines an overt speech production task in AWS and fluent speakers (FS) using high-temporal resolution magnetoencephalography (MEG) measurements of movement preparation and execution related brain oscillations (Cheyne, 2013).

Brain oscillations are a manifestation of the recruitment of neural resources for the performance of any cognitive task. They are proposed to be the primary mechanism by which the brain transfers and processes information and reflect changes in the degree of synchrony between interactions of local neural networks (Varela et al., 2001; Buzsáki and Draguhn, 2004; Fries, 2005). A cognitive task, or event, can trigger an event-related synchronization (ERS), which reflects an increase in the power of oscillatory activity, or an event-related desynchronization (ERD), which reflects a suppression (i.e., a decrease of power) of oscillatory activity. Pertinent to this article is the widely observed suppression of beta oscillations (15–30 Hz) in the motor cortex occurring prior to voluntary motor movement and beginning as early as 1 s prior to movement onset (Pfurtscheller and Da Silva, 1999; Bai et al., 2005; Kilner et al., 2005; Alegre et al., 2006; Jurkiewicz et al., 2006; Tzagarakis et al., 2010; Kilavik et al., 2013). Studies have proposed that pre-movement suppression of beta power reflects the preparation for the motor response, or translation to motor movement parameters, and can be modulated by task difficulty (Pfurtscheller and Da Silva, 1999; Cheyne, 2013; Kilavik et al., 2013). In contrast, an increase of beta power in the motor cortex is proposed to play a role in maintaining the current motor state and inhibiting the initiation of new motor plans (Pfurtscheller and Klimesch, 1991; Pfurtscheller and Da Silva, 1999; Neuper and Pfurtscheller, 2001; Engel and Fries, 2010). These studies suggest that beta oscillations play a critical role in the facilitation of speech and non-speech motor movement. In contrast, the suppression of power in the lower alpha range (8–13 Hz) has been widely observed in visual and somatosensory regions engaged during task-specific processing and in this way alpha suppression has become a recognized index of disinhibition of task-relevant regions and a correlate of brain activation (Pfurtscheller and Da Silva, 1999; Klimesch et al., 2007; Klimesch, 2012).

Recent studies found that the preparation for speech production induces changes in alpha and beta oscillations across sensory and motor regions (Salmelin et al., 2000; Saarinen et al., 2006; Gehrig et al., 2012; Alho et al., 2014; Jenson et al., 2014; Liljeström et al., 2014). In Gehrig et al. (2012), a visual cue was displayed to signal participants, whether the upcoming sentence sequence will have to be read overtly or covertly. The authors found that the preparation for overt reading induced strong alpha suppression in the left temporal lobe (BA21, 22, 41), and beta suppression in the bilateral parietal lobe (BA 5, 7, 40) and left the articulatory motor region (BA4) beginning at 350 ms after the preparation cue was given (Gehrig et al., 2012). In a syllable overt-production task, Jenson et al. (2014) also found an induced suppression of beta (~20 Hz) and alpha (~10 Hz) peak activity in the left premotor (BA6) and primary motor cortex (BA4) beginning 300 ms after the cue to speak (Jenson et al., 2014). Beta suppression in the motor cortex during speech preparation has been proposed to reflect a feed-forwarding of the speech plan to the motor effectors and to the sensory regions required for monitoring speech output, while alpha suppression of the auditory regions was proposed to reflect the priming of auditory feedback loops required for speech production (Crawcour et al., 2009; Engel and Fries, 2010; Cuellar et al., 2012; Gehrig et al., 2012; Klimesch, 2012; Bowers et al., 2013; Kilavik et al., 2013; Liljeström et al., 2014). Preparation for speech production was also shown to induce global neural coherence in the high beta range (25–31 Hz) between bilateral primary motor and premotor cortices, and the inferior and middle temporal gyri in the auditory cortex (Alho et al., 2014; Liljeström et al., 2014), thereby demonstrating the complex interconnections within the speech-motor network prior to speech onset.

An element of the motor preparation for speech production in AWS was quantified in a study that delivered a transcranial magnetic stimulation pulse to the bilateral orofacial motor cortex prior to the onset of speech production (Neef et al., 2015). This study found that prior to speech onset AWS show reduced excitability in the left orofacial primary motor cortex compared to FS. The authors proposed that AWS lack sufficient sensory-motor planning of speech gestures in a region of the motor cortex that is a key player in establishing such programs (Bohland et al., 2010; Guenther and Vladusich, 2012; Neef et al., 2015). Other authors have suggested that speech preparation in AWS is incomplete because auditory-sensory feedback targets are improperly set-up (Beal et al., 2010; Cai et al., 2014a; Daliri and Max, 2015). For example, in a delayed reading task, Daliri and Max (2015) recorded the auditory N100 response to probe tones during the speech preparation stage. The study found that while FS showed the expected N100 suppression, AWS did not (Daliri and Max, 2015). The auditory N100 suppression prior to speech onset is proposed to reflect the preparation of the auditory cortex for the efferent copy of the motor speech plan in order to allow for proper monitoring of speech output (Curio et al., 2000; Gunji et al., 2001; Houde et al., 2002; Heinks-Maldonado et al., 2005; Martikainen et al., 2005; Flinker et al., 2010). Daliri and Max (2015) interpreted their findings as a deficient auditory prediction of speech output that interrupts monitoring and smooth execution of speech plans in AWS.

Measuring neural oscillatory changes in the speech-motor network of AWS during speech production tasks allows for a more natural way to study the assembly of the speech network compared to using probe tones or external stimulation. A recent study found that at rest (i.e., no task), AWS showed decreased inter-hemispheric functional connectivity in the beta (13–30 Hz) and low gamma (31–44 Hz) range between the bilateral inferior frontal gyri (BA44, 45) and the premotor and motor areas (BA4, 6), when compared to controls (Joos et al., 2014). Considering that communication between these regions is critical for the formulation of the motor plan (Guenther et al., 2006). Joos et al. (2014) proposed that reduced neural connectivity between these regions at rest may cause a de-synchronization of articulatory muscle groups once speech is initiated, which may contribute to a stuttering moment. Studies involving speech tasks in AWS have primarily focused on quantifying the speech production stage following the audible speech onset. During a reading task, AWS showed increased beta oscillations across the cortex, particularly in the right temporo-perietal lobe (Rastatter et al., 1998). Salmelin et al. (2000) found that rather than showing exaggerated beta activity, AWS tended to have a more right-lateralized suppression of beta power in the mouth motor cortex during single-word reading, while FS showed a primarily left-lateralized pattern (Salmelin et al., 2000). Similar right laterality was observed in decreased alpha and beta oscillations during rest in children who stutter, which was proposed to reflect reduced cortical maturation (Özge et al., 2004). Some authors have argued that beta oscillations reflect a component of a wider internal timing network that coordinates brain neural responses based on temporal processing (Etchell et al., 2014a,b). According to Etchell et al. (2014a,b), aberrant modulation of cortical beta power in AWS is compensating for anomalies in the internal timing network, supported within sub-cortical loops (i.e., basal ganglia).

There is thus sufficient evidence to demonstrate that cortical neural oscillations are implicated in stuttering, with varying hypotheses as to their exact role. Although past studies have not examined speech preparation *per se*, it is conceivable that the differences observed during rest and speech execution in the afore-mentioned studies will also extend to the speech-motor preparation stage. Indeed, when Sowman et al. (2012) compared neural oscillations that preceded the onset of stuttered and fluent utterances in a single stuttering subject, they found that fluent utterances followed a stronger and earlier activation (in the 1–45 Hz range) of the left inferior frontal gyrus (BA47), which occurred as early as 500 ms prior to speech onset (Sowman et al., 2012). The inferior frontal gyrus is proposed to be a critical region in the integration of sensory and motor information (Kell et al., 2009; Papoutsi et al., 2009; Hickok, 2012). This study illustrated the importance of separating processes occurring prior to speech onset (i.e., “preparation”) from those occurring following it (i.e., “execution”). Such a separation would be critical to understanding differences in oscillatory modulation prior to a speech disruption, compared to fluent production.

The primary objective of the current work is therefore to compare the neural modulation in motor and sensory regions of the speech-motor network during preparation for *and* execution speech between AWS and FS. The task design and analysis allowed us to separate the speech preparation stage from speech execution. Consequently, we were able to observe differences in the speech-motor network of AWS and FS preceding speech onset.

## Materials and Methods

### Participant Criteria

Twelve AWS (mean age 30 years, range 21–45, 2 females) and 12 control FS (mean age 32, range 21–43, 2 females) were recruited. Consent was obtained as approved by the Hospital for Sick Children (Toronto) and University of Toronto Research Ethics Boards. All participants were right-handed, as determined by the Edinburgh Handedness Inventory (Oldfield, 1971), had no self-reported neurological conditions affecting motor ability, speech, vision, or hearing and reported that English was their primary language of use. Additionally, the FS group reported no history of speech or language therapy, while the stuttering participants reported no history of speech or language therapy other than stuttering-specific therapy. To be included in the stuttering cohort, participants must have scored at least “mild” on the Stuttering Severity Index (SSI-4; Riley, 1972; Riley and Bakker, 2009) and self-reported to have been stuttering since early childhood. One participant showed no stuttering behavior during Visit 1 and therefore received a score of 0 on the SSI-4. However, this participant was enrolled in the study following a confirmation of their stuttering behavior from the Speech and Stuttering Institute, Toronto. Control participants were selected to match the stuttering participants by age and sex.

### Stimuli Selection

Single words were generated from the English Lexicon Project (Balota et al., 2007), a commonly used database for stimulus generation (Keuleers et al., 2010; Brennan et al., 2014; Plummer et al., 2014). Controlled variables were filtered for syllable number (2 characters), phoneme length (5–7 characters), letter length (5–9 characters), bigram frequency sum (18,000–28,000), word frequency (1–20 per million), and naming reaction time (set to a maximum of 630 ms). The filters generated a list of 471 words. From this list morphemic derivatives and proper nouns were removed, as well as words eliciting general and stuttering-specific threats, as suggested by recent studies (Hennessey et al., 2014; van Lieshout et al., 2014). The final list consisted of semantically neutral 414 words.

### Visit 1: Word Ranking Task

In the first study visit, AWS were asked to rank each word from the 414 word list on how likely they are to stutter on it in a spontaneous conversation setting. Each word from the randomized 414 word list was presented one at a time at the center of a computer screen. Participants were asked to press a number one through six to indicate their ranking (1 = “Extremely unlikely”, 2 = “Very unlikely”, 3 = “Somewhat unlikely”, 4 = “Somewhat likely”, 5 = “Very likely”, 6 = “Extremely likely”), after which the next word was presented. Similar methods of identifying stuttering-prone words have been applied in previous studies (Bowers et al., 2012; den Ouden et al., 2014). In order to provide the FS group with the same familiarization with the word-list, FS performed a similar ranking task, but were asked instead to rate each word on the likelihood that they have used it during the previous week. The same ranking scale was used. As this was purely a familiarization task for the FS, their rankings were not used in the study. Both groups performed the ranking task twice using a randomized word order. The Vocabulary Knowledge and Digit Span sub-tests from the Wechsler Adult Intelligence Scale-III (WAIS-III; Wechsler, 1997) were administered to both groups between the two ranking tasks. These subtests were used to confirm that there were no group differences in vocabulary or working memory (Table 1). For the AWS group only, the WAIS-III was followed by the SSI. The word ranking process was used in order to identify 110 high and 110 low likelihood of stuttering words for each participant, selected according to ranking order. However, for the purpose of the current investigation, no differentiation was made between the word rankings and all 220 words were analyzed together for each participant. Analysis of the effect of stuttering anticipation ranking on speech preparation is currently under-going. This trial number exceeds the recommended minimum of 100 trials suggested in the MEG literature (Gross et al., 2013).

**Table 1:** **Mean (SD) of measured variables in Visit 1**.

	AWS	FS	*p*-value
Participants	12	12	–
Sex	10 M, 2F	10 M, 2 F	–
Age	32 (6)	30 (8)	0.46
Vocabulary	12.4 (2.4)	13.0 (2.1)	0.5
Digit Span	11.3 (2.9)	12.4 (2.1)	0.26
STAI—State	32.4 (11.9)	28.9 (8.9)	0.42
STAI—Trait*	40.1 (8.6)	32.7 (7.5)	0.02
STAI—Total	73 (19)	62 (16)	0.13

*Significant group differences are indicated by the asterisk next to the variable name*.

### Visit 2: MEG Task

Participants completed the imaging experiment within 11–90 days of their ranking-task session, as scheduling permits. Each participant’s specific list of 220 words was visually presented for cued overt speaking during the MEG scan. The words were presented in random order and appeared at the onset of the carrier phrase “[X] is a word”. Each FS received the same words as their matched adult who stutters.

**Figure 1:**
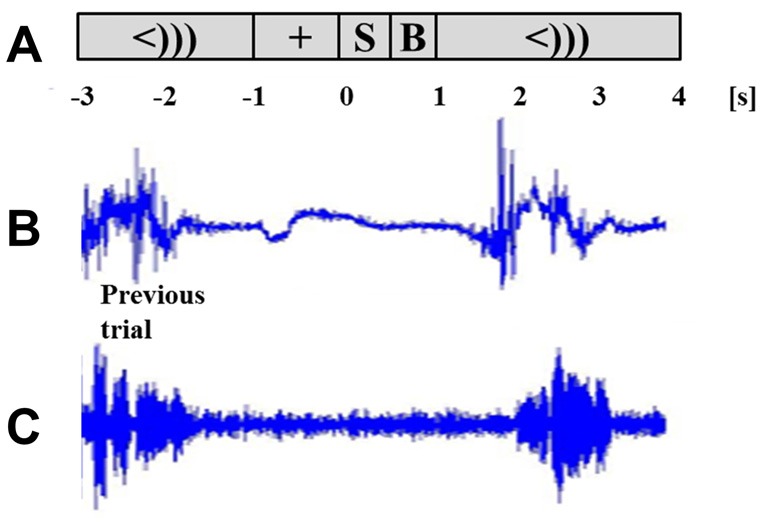
**Task schematic and time course of two successive trials.** Task sequence **(A)** includes a fixation (+) of alternating length (1 or 2 s), stimulus sentence (S, 0.5 s), a blank screen (B, 0.5 s) and the cue to speak (<))), 3 s). Lip EMG signal **(B)** and voice signal **(C)** are taken from one participant. The response of the previous trial and the succeeding trial are shown for the chosen participant.

Participants were acquainted with the task using a sequence of eight test words prior to being placed in the MEG. Participants were instructed that if stuttering were to occur, they were to complete the entire utterance, even if it runs into the next trial. Trials that were contaminated with a speech from the previous trial were removed during the data processing stage (see “Data Processing” Section). The task sequence was presented while participants were seated upright. As shown in Figure 1, each trial started with a target fixation cross in the middle of the screen that was presented for a randomly alternating duration of 1 or 2 s. The stimulus (“[X] is a word”) then appeared for 500 ms, followed with a 500 ms blank screen. A cue immediately followed in the form of “<)))” and remained for 3 s. Participants were to speak the stimulus sentence following the speech cue. A cued overt repetition task allowed for a better separation of the speech preparation and speech execution windows. The cue was followed by the fixation cross of the next trial. The experiment ran in 55 trials per block, for four blocks. At the end of every block, a 5-s message was displayed to notify the participant of their progress. All presented text was in white Arial font, height 1.7 cm, centered on a gray back-projection screen 75 cm away. All participants had normal vision and could read the stimuli. Stimulus presentation was performed using PsychoPy software^1^.

### Data Acquisition

Neuromagnetic activity was recorded continuously using a 151-channel whole head CTF (Omega) system located at the Hospital for Sick Children in Toronto, Canada. All data signals were collected at 12,000 samples/s and band passed at 0–4000 Hz to preserve acoustic integrity of the speech signal. MEG data were filtered off-line to 0.4–250 Hz and down-sampled to 1000 samples/s. Verbal responses were recorded using a Rode NTG-2 directional condenser microphone placed about 1.8 m from the participant and recorded as an auxiliary channel. Stimulus onset was indicated by a luminance sensor on the back of the projection screen to correct for signal delay between the presentation computer and the display. Participants were asked to avoid blinking between the stimulus and the start of their speech and to minimize head movement during speech production. Trials were later screened for artifacts prior to data analysis (described in Section “Data Processing”). Participant head position was measured continuously during the MEG recordings using fiducial coils placed at the right and left pre-auricular and nasion points for later co-registration with the anatomical MRI. Immediately following the MEG session a T1-weighted structural MRI MPRAGE gradient echo sequence (flip angle = 9°, TE/TR = 2.96 ms/2300 ms, 192 sagittal slices, 1 mm thick, 256 × 256 matrix, 25.6 cm FOV) was acquired for each participant on a 3T Siemens Trio MRI scanner at the Hospital for Sick Children.

### Reliability of Severity and Stuttering Measures

The speech of the AWS group was monitored offline by the first author in order to determine stuttering incidences. Each trial was rated as “yes”, “no” or “maybe” for containing a stuttered moment. A second independent rater, a registered speech language-pathologist, performed the same monitoring on three AWS participants. A trial was identified as stuttered if classified by one rater as a “yes” and the second rater as either a “yes” or a “maybe”. The level of agreement between the two raters varied from 90% to 96% across the three evaluated participants. The number of ambiguous trials (a rating of “maybe” from both raters) did not exceed 7%. The speech-language pathologist also re-calculated the SSI score independently for four AWS. Final SSI inter-rater differences ranged from 0 to 5 points, but all severity scores remained within the same severity category.

### EMG Recording

Surface EMG was measured from the orbicularis oris muscle bilaterally using two pairs of bipolar EMG electrodes (AMBU adhesive electrodes, oval design, 22 × 30 mm) above and below the lip (Salmelin et al., 2000; Goncharova et al., 2003; Saarinen et al., 2006; Liljeström et al., 2014). Each pair of electrodes (upper and lower lip) was amplified using differential input and the final subtracted signal for the right and left electrodes underwent the same processing as the MEG channels (12,000 samples/s, band passed at 0–4000 Hz, filtered 0.4–250 Hz, down-sampled to 1000 Hz). Speech onsets and offsets were identified and marked offline after rectifying the 0.4–250 Hz signal (Salmelin et al., 2000; Salmelin and Sams, 2002). An automated script was used to identify onsets at one standard deviation (SD) from baseline. Only one channel was selected for onset marking, and was selected based on a visual inspection for a cleaner signal.

### Data Processing

Continuous MEG data were segmented off-line into 8-s epochs (or trials). One epoched dataset was time-locked to the presentation of the stimulus (“stimulus-locked”), and the other was time-locked to lip-movement onset determined from the EMG signal (“speech-locked”), all within a −4 to 4 s time-window. One control participant had only 145 trials recorded due to technical problems during the scan, while all others had 220 trials. Of a total of 2640 trials across 12 AWS, 344 (13%) stuttered trials were identified across nine AWS according to the reliability measures described in Section “Reliability of Severity and Stuttering Measures”. All stuttered trials were removed from this study for the purpose of analyzing perceptually fluent speech production in AWS. Trials in each data set were further visually inspected and removed if the following was observed: (a) fixation period was contaminated with voicing or EMG signal from the previous trial; (b) EMG artifacts were observed between stimulus presentation and speech onset or during fixation; and (c) if MEG activity exceeded 5 picoTesla (typically corresponding to eye blinks or muscle artifact). This inspection was performed for stimulus-locked and speech-locked epoched datasets separately. No trials were removed due to excessive head movement (>1 cm). Across all participants, only 30 trials showed head movement between 0.6 and 0.8 cm, and for one participant only, 80 of 220 trials were between 0.7–1 cm. The head movement in all remaining trials was below 0.6 cm. The final average trial numbers for the AWS and FS groups, respectively were 160 and 193 across both datasets (min = 87, max = 217 across both groups), but there was no significant group difference in trial number. Within-group one-sample and two-sample *t*-tests confirmed there was no difference in trial number between the two data sets.

### SAM Beamformer Analysis

Source analysis of frequency specific power changes was conducted using the Synthetic Aperture Magnetometry (SAM) algorithm (Robinson and Vrba, 1998) implemented in C++ and Matlab (Mathworks, Natick, MA, USA) using the *BrainWave* toolbox^2^. SAM images of induced power changes over time were generated by subtracting baseline source power activity from a sliding active time window of 200 ms duration defined at 50 ms intervals (starting from stimulus onset to 1400 ms in the stimulus-locked data and from 1200 ms prior to EMG onset to 200 ms post EMG onset in the speech-locked dataset). A fixed 200 ms window during the fixation period (−500 to −300 ms preceding stimulus onset) was used as baseline. The baseline window was visually inspected once averaged across all participants to confirm the absence of time-locked eye-blinks. Pseudo-T images were computed for alpha (8–13 Hz) and beta (15–25 Hz) frequency bands over the entire brain at 4 mm resolution. Resulting SAM pseudo-T images were averaged across participants and spatially normalized to the Montreal Neurological Institute (MNI) template using SPM8^3^. MNI coordinates were converted to Talairach coordinates using the mni2tal conversion in reference to the Talairach brain atlas anatomical locations^4^. Talairach coordinates were used for reporting all localizations. To improve anatomical visualization, SAM beamformer results were interpolated onto high-resolution cortical surfaces which were generated using the CIVET image-processing pipeline developed at the MNI (Kim et al., 2005), which provides detailed cortical surface meshes for cortical source reconstruction using a fixed number of vertices (by default 81,920 vertices per hemisphere).

### Statistical Analyses

For both datasets, coordinates of maximal source power change were used to extract single-trial source activity (virtual sensors) over a period of −4 to +4 s relative to stimulus-locked baseline (−0.5 to −0.3 s) using the voxel of maximal activation in each participant’s non-normalized SAM image. Individual peaks were localized within a 10 mm search radius from the group peak (Cheyne et al., 2006). Time series were band-pass filtered between 1 and 100 Hz and used to generate time-frequency representation (TFR) plots using a Morlet wavelet based decomposition (wavelet cycles = 5, frequency step = 1 Hz). TFR plots were inspected to assess changes across frequency bands. Power was averaged across each frequency range of interest to generate an envelope time-course of ERD or ERS in the frequency bands of interest. In the following sections the terms ERD, suppression, or power decrease are used interchangeably.

Participant-specific ERD onsets and ERD offset times were used to compare the initiation and termination of the oscillatory changes across groups. An ERD onset was defined as a first time-point below zero amplitude change following the baseline fixation (see Figure 4, Section “Quantifying Beta (15–25 Hz) Power Decrease and Increase in the Mouth Motor Cortex (BA6) During Preparation and Execution of Speech”). The ERD offset was determined at the time when the time-course returned to zero after the ERD onset. If the time-points could not be determined for a participant, the participant was not included in the group average.

In addition, two stages of the speech task were defined for analysis: (a) speech preparation and (b) speech execution. The speech preparation stage was defined from stimulus presentation to the speech cue (0–1 s) in the stimulus-locked dataset. This defined the period during which participants saw the sentence stimulus and awaited the cue to speak it out loud. The speech execution stage was defined from the speech onset, determined from the lip EMG signal in the speech-locked dataset (0 s), to the participant-specific ERD offset time (see Figure 4, Section “Quantifying Beta (15–25 Hz) Power Decrease and Increase in the Mouth Motor Cortex (BA6) During Preparation and Execution of Speech”, for a depiction of these time windows). The degree of suppression was quantified by integrating the time-courses of the source power relative to baseline power (in units of dB) in these two specified windows and averaging across participants.

Latencies and integrated power values were analyzed using ANOVAs, Tukey Honest Significant Difference (HSD) multiple comparison test, and standard *t*-tests. Pearson correlations and multiple linear regressions were used to investigate variable relationships. The same analysis was applied to quantify ERS patterns.

## Results

### Participant Response Times

Response times were computed from the stimulus-locked data where the response was defined as the time of the EMG signal relative to the cue to speak (at 1 s). Group average response times were 0.230 s (SD: 0.083 s) and 0.225 s (SD: 0.103 s) for AWS and FS respectively, with no significant group difference (*p* > 0.5, one-sample *t*-test).

### Localization of Alpha (8–13 Hz) and Beta (15–25 Hz) Power Changes

**Figure 2:**
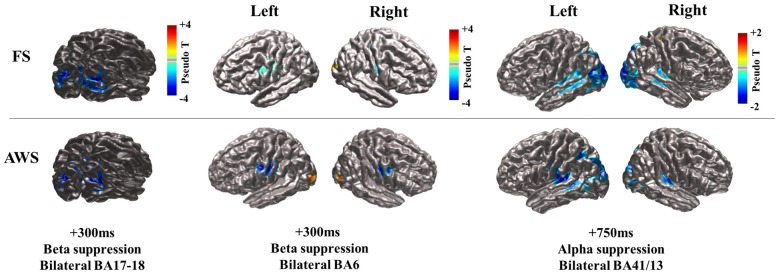
**CIVET-generated surface images with imposed Synthetic Aperture Magnetometry (SAM) localization of beta and alpha suppression (blue) during speech preparation (stimulus-locked) compared between fluent speakers (FS) and adults who stutter (AWS).** The time at which peak magnitude was observed relative to stimulus presentation is indicated. The bilateral cuneus is displayed for the beta suppression only, but localizations were the same for the observed alpha suppression in that region (see Table 2).

Stimulus presentation induced power changes in the bilateral cuneus (BA18, 17), precentral gyrus (BA6), and the bilateral posterior insula and superior temporal gyrus (BA 13, 41). The bilateral cuneus showed a power decrease in the beta and alpha ranges, immediately following stimulus presentation. The precentral gyrus showed exclusively beta power decrease, while the posterior insula and superior temporal gyrus showed a distinct alpha power decrease. The localized group average co-ordinates for these regions are listed in Table 2 along with group peak values (Pseudo-T). The localized coordinates of beta power decrease in the BA6 region were in close proximity to the mouth motor cortex (Takai et al., 2010; Grabski et al., 2012), while the coordinates of the alpha power decrease in the BA13 and BA41 regions were in close proximity to the primary auditory cortex (Weeks et al., 2000; Rademacher et al., 2001; Desai et al., 2005; Kopco et al., 2012; Wasserthal et al., 2014). Figure 2 displays the activity found in all three regions bilaterally relative to stimulus presentation, imposed on the generated surface images. Although Figure 2 shows localizations identified in the stimulus-locked dataset, the same peaks were observed in the speech-locked dataset in the window preceding speech onset. A two-sample *t*-test on the localized *x*, *y*, and *z* coordinates across all participants confirmed there was no significant difference between the stimulus and speech-locked datasets for any of the localized regions (*p* > 0.2). This allowed the comparison of the two datasets in the analysis described in Section “Quantifying Beta (15–25 Hz) Power Decrease and Increase in the Mouth Motor Cortex (BA6) During Preparation and Execution of Speech” below. The average over participant-specific coordinates was in good agreement with the SAM localized group peaks suggesting that there was not a single participant that was skewing the SAM localization of the group average peak. The occipital region is left out of the presented analysis.

**Table 2:** **Talairach coordinates (mm) of Synthetic Aperture Magnetometry (SAM) group averaged peak amplitude (Pseudo-T) for alpha and beta suppression preceding speech onset**.

	**AWS**		**FS**	
Anatomy	*X, Y, Z*	Pseudo-T	*X, Y, Z*	Pseudo-T
**Beta**
Left BA6	−46, −2, 28	−4.6	−46, −2, 28	−3.14
Right BA6	50, 2, 31	−3.4	50, 2, 31	−1.35
Left BA18	−18, −77, 17	−4.53	−18, −81, 21	−4.11
Right BA18	22, −73, 17	−4.99	22, −81, 21	−4.86
**Alpha**
Left BA13/41	−32, −44, 22	−2.09	−53, −23, 10	−1.31
Right BA41	53, −19, 14	−1.5	50, −23, 7	−1.01
Left BA18	−14, −81, 17	−3.82	−22, −84, 21	−4.03
Right BA18	22, −77, 20	−3.73	14, −85, 13	−4.3

### Time-Courses of Alpha (8–13 Hz) and Beta (15–25 Hz) Power Changes in the Stimulus-Locked and Speech-Locked Datasets

**Figure 3:**
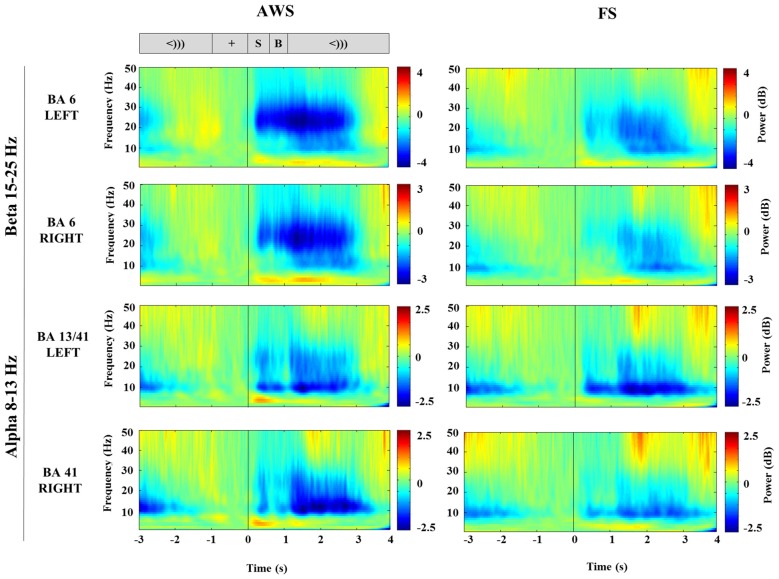
**Stimulus-locked time-frequency representation (TFR) plots of virtual sensors extracted from the bilateral mouth motor cortex (BA6) and auditory-sensory cortices (BA13/41) compared between AWS and FS.** The corresponding task sequence time-line is displayed at the top for orientation and includes the fixation (+), stimulus sentence (S, 0.5 s), a blank screen (B, 0.5 s) and the cue to speak (<))), 3 s). Suppression (blue) is apparent following the stimulus presentation (S).

**Figure 4:**
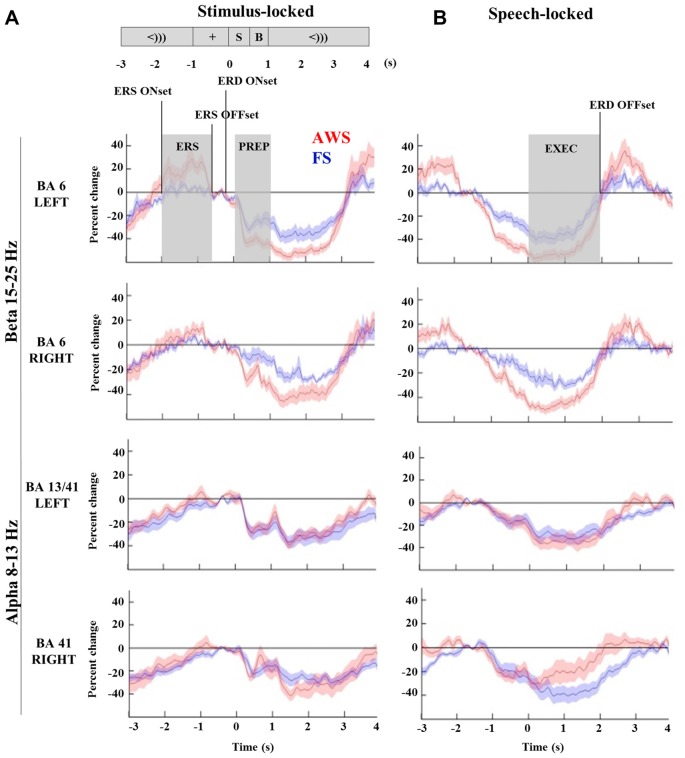
**Time-courses of beta (15–25 Hz) and alpha (8–13 Hz) suppression in the bilateral mouth motor cortex (BA6) and auditory regions (BA41, 13), respectively, compared across AWS and FS.** All windows and time-points of interest are indicated on the top plot. Speech preparation (PREP) was defined from stimulus presentation to the speech cue (0–1 s) in the stimulus-locked dataset **(A)**. Speech execution (EXEC) was defined from the speech onset to participant specific beta event-related desynchronization (ERD) offset time in the speech-locked dataset **(B)**. A window of beta synchronization (event-related synchronization, ERS) is indicated preceding the stimulus presentation, as well as the corresponding ERS onset and offset times (**A**, top). ERD onset time is marked for the purpose of the latency analysis in Section “Group Comparisons of Alpha and Beta Power Suppression Latencies”.

TFRs were created from Morlet wavelet decomposition applied to the single trial virtual sensor data for the localized motor and auditory peaks. The TFRs illustrate the frequency bands where oscillatory power changes were observed. As seen in Figure 3, the signal extracted from the localized BA6 shows a power decrease in the 15–25 Hz beta range (blue), while the signal extracted from the BA13/41 regions shows a power decrease in the lower 8–13 Hz alpha range. The time courses across these frequency bands are plotted in Figure 4. In the stimulus-locked time-courses, suppression of beta and alpha power is induced as soon as the stimulus sentence is presented (0 s) (Figure 4A), and continues to decrease as the participants await the cue to speak. Similarly, the speech-locked dataset also showed that the suppression of alpha and beta power in the auditory and motor regions significantly preceded the actual speech-onset at 0 s (Figure 4B). An unexpected finding was and additional beta power increase in the stimulus-locked time-course of the left BA6, which was particularly strong in the AWS group (Figure 4A). This appears as the increase in percent change between –2 and –1 s, preceding the stimulus presentation.

### Quantifying Beta (15–25 Hz) Power Decrease and Increase in the Mouth Motor Cortex (BA6) During Preparation and Execution of Speech

To quantify differences in the magnitude of alpha and beta power changes, the source power was integrated to obtain the area under the curve for the speech preparation and execution stages. Speech preparation (*PREP*) was defined from stimulus presentation to the speech cue (0–1 s) in the stimulus-locked dataset. Speech execution (*EXEC*) was defined in the speech-locked dataset to begin from the speech onset (0 s) to participant specific *ERD offset* time. The power increase observed in the beta range (*ERS*) was integrated over the curve of the window defined by each participant’s ERS onset and offset times. All relevant time points are indicated in Figure 4 (top left) and were defined in the same manner for both alpha and beta analyses.

**Figure 5:**
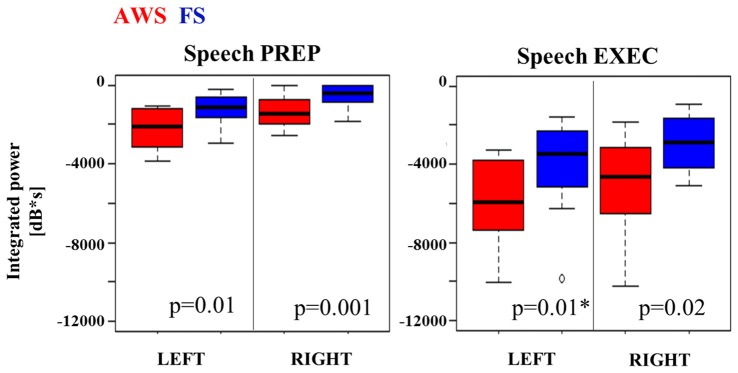
**Integrated beta suppression during speech preparation (PREP) and execution (EXEC).** *Note*. AWS group showed stronger left beta suppression during speech execution once the outlier was removed (*).

The integrated beta suppression value was compared across group, hemisphere and stage (preparation, execution) in a three-way ANOVA. Significant main effects were observed for hemisphere (*p* = 0.008), group (*p* < 0.0001) and speech stage (*p* < 0.0001), but no significant interactions were found. A multiple comparison correction test (Tukey-HSD) confirmed that the speech execution stage involved significantly greater suppression overall when compared to the preparation stage, which was true for each hemisphere in each of the groups (*p* < 0.001). This was expected as beta suppression was maintained at times up to 3 s past speech onset. The primary contrast of interest was the difference between groups when compared within the same hemisphere and speech stage. Although these contrasts did not survive Tukey-HSD correction they are displayed in Figure 5 with the corresponding two-sample *t*-test results. AWS showed stronger beta suppression in the bilateral mouth motor cortex. This effect was already apparent during the speech preparation stage, and persisted in the execution stage.

**Figure 6:**
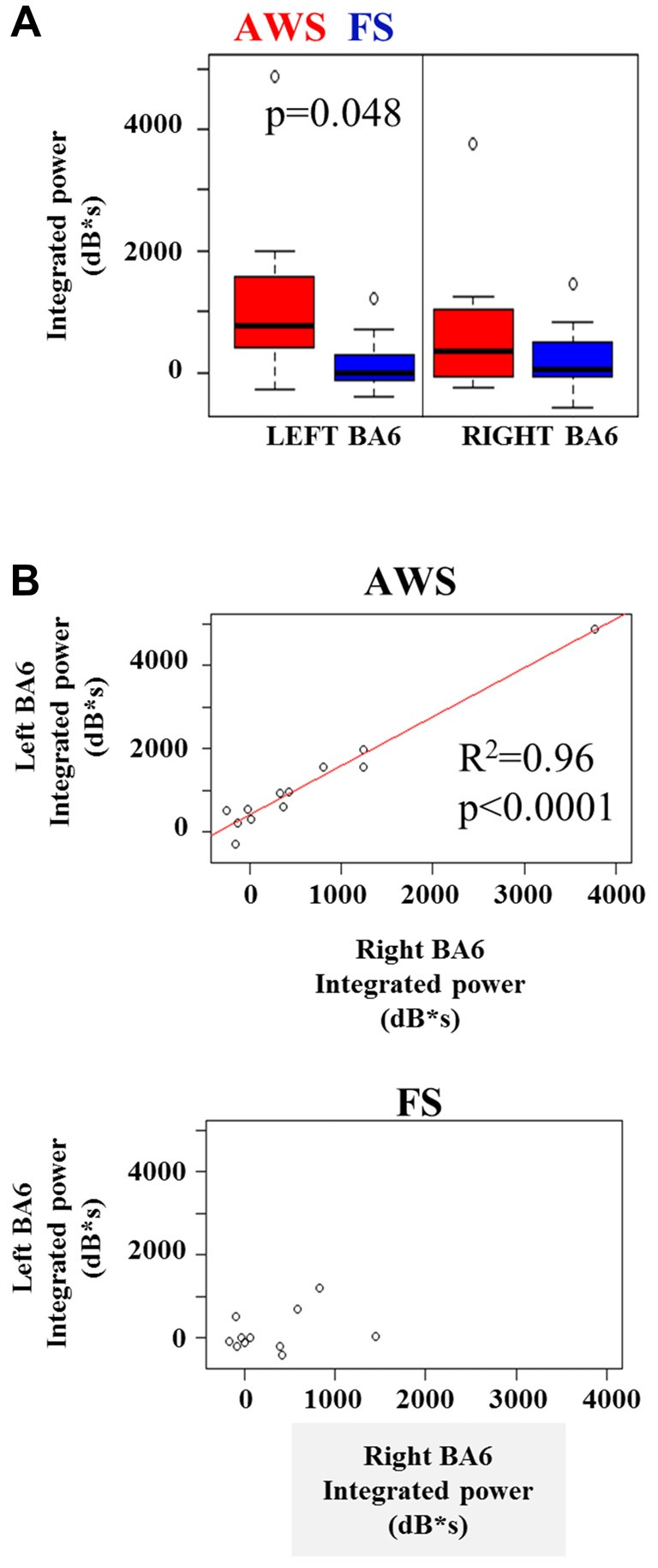
**(A)** Integrated beta ERS power in the left and right mouth motor cortex (BA6) preceding stimulus onset. **(B)** Significant linear relationship between the integrated beta ERS in the left and right BA6 of AWS (top). No significance found in FS (**B**, bottom).

A two-way group by hemisphere ANOVA of the integrated beta synchronization found a significant main effect of group (*p* = 0.01). Multiple comparison correction revealed a moderately stronger increase in beta power in the left BA6 of AWS compared to FS (*p* = 0.048, Figure 6A), but no significant difference was found in the right BA6. However, a strong correlation was observed between the pre-stimulus beta power increase in the left and right BA6 of AWS (*R*^2^ = 0.96, *p* < 0.0001), which was not observed for FS (*R*^2^ = 0.33, *p* > 0.05; Figure 6B). It is noteworthy that the significant correlation survived the removal of the top right-most point (*R* = 0.83, *p* < 0.001, data not shown). No such correlation was observed for the beta suppression.

### Quantifying Alpha (8–13 Hz) Power Decrease in the Posterior Temporal Cortices During Preparation and Execution of Speech

The three-way (group, hemisphere and stage) ANOVA was repeated on the integrated alpha suppression extracted from the BA13 and BA41 time-courses. A significant effect was found only for speech stage, with alpha suppression increasing bilaterally from the preparation to the execution stage (*p* < 0.00001), corresponding to the expected prolonged auditory engagement during speech production. However, no group or hemisphere differences survived multiple comparison correction or two-sample *t*-tests (data not shown). It is noteworthy that Figure 4 (bottom, right) appears to show a group difference in the time-courses of the right BA41. It is possible that due to variability in the data, the *t*-test was underpowered to detect this difference. There were therefore no significant group differences in induced alpha power change in the auditory regions during the speech task.

### Predictions of Stuttering Severity

**Figure 7:**
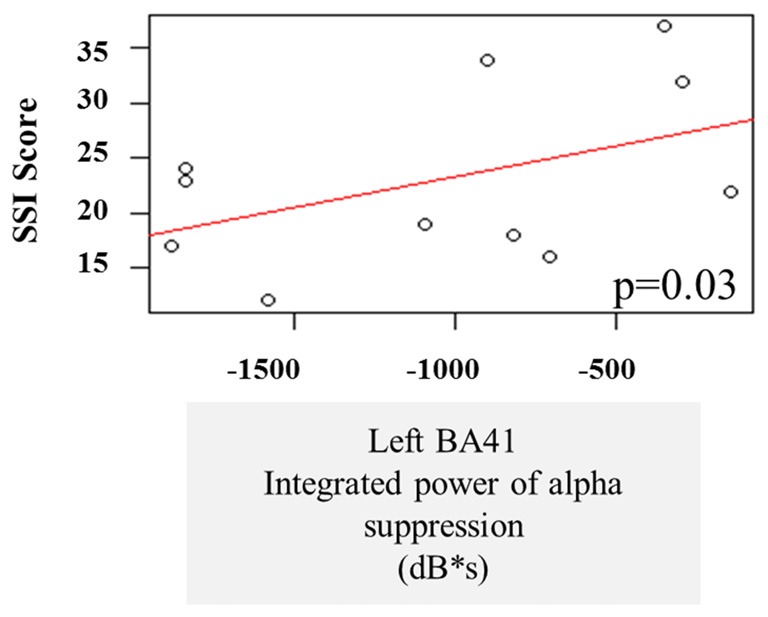
**Linear regression of stuttering severity on alpha suppression in left primary auditory cortex (BA41)**.

A multiple linear regression was performed on the integrated alpha and beta power suppression values to see whether the observed oscillatory changes during the speech preparation or execution stage can be predictive of stuttering severity. One AWS participant was not included in these correlations because he received an SSI score of 0 during Visit 1 and this was not considered to be representative of their stuttering behavior (see “Participant Criteria” Section). There was no significant effect of beta suppression in the left and right BA6 on the stuttering severity, and no interaction. There was also no significant effect of pre-stimulus beta power increase in the SSI score. However, we note that there was a main effect of alpha power suppression in the left temporal gyrus (BA41) on the SSI during the speech preparation stage (*p* = 0.03). The three severe AWS participants (SSI > 30) tended to have less alpha suppression in the left posterior temporal regions during speech preparation (Figure 7).

### Group Comparisons of Alpha and Beta Power Suppression Latencies

**Figure 8:**
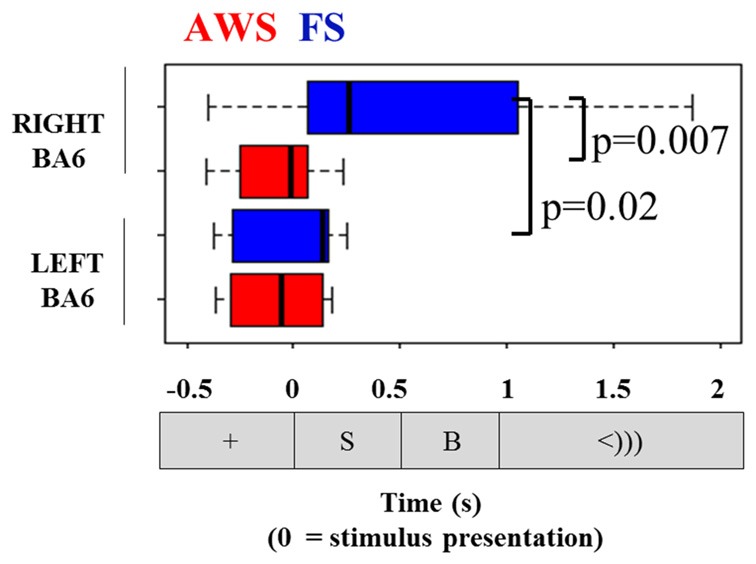
**Onset of beta suppression in the left and right mouth motor cortex (BA6), relative to stimulus presentation.** Task sequence time-line is displayed for orientation.

Comparisons of the timing of the alpha and beta suppression time-courses were carried out using suppression onset time (*ERD onset*), defined relative to the stimulus-locked dataset, and suppression offset times (*ERD offset*), which was defined relative to the speech-locked dataset (see Figure 4). A group by hemisphere ANOVA found a main effect of group (*p* = 0.01), main effect of hemisphere (*p* = 0.025) and a moderately significant group by hemisphere interaction (*p* = 0.03). A multiple comparison test (Tukey-HSD) revealed that while there were no group differences in the ERD onset of the left BA6, AWS recruited the right BA6 significantly earlier than FS (*p* = 0.007). AWS recruited both the right and left BA6 simultaneously, while FS first predominantly recruited the left BA6 and followed by the right much closer to speech onset time (*p* = 0.02). These results are depicted in Figure 8. No differences were observed in the ERD offset times. The analysis was repeated for alpha suppression latencies from the bilateral auditory regions. No significant main effects were observed.

## Discussion

The current study is the first to characterize neural alpha and beta oscillatory modulation associated with the recruitment of the speech-motor network during speech preparation and execution in AWS. The adopted cued overt speech task allowed the separation of what is presumed to be preparation and execution stages of speech production by time-locking the data to either stimulus presentation or speech onset, respectively. We have found that activation of the bilateral visual, motor, and auditory components of the speech network was initiated as soon as the articulatory sequence was displayed and significantly preceded speech onset itself. The bilateral cuneus (primary visual cortex) displayed both alpha (8–13 Hz) and beta (15–25 Hz) power suppression, the mouth motor cortex displayed primarily beta (15–25 Hz) power suppression, and the posterior-temporal regions, in close proximity of the primary auditory cortex, displayed primarily alpha (8–13 Hz) power suppression. The primary finding in this study was that compared to FS, AWS showed significantly stronger beta suppression in the bilateral mouth motor cortex during speech preparation (i.e., preceding speech onset), and during speech execution (i.e., following speech onset). Furthermore, comparisons of the beta suppression onsets revealed that the right mouth motor cortex was recruited significantly earlier in AWS compared to FS. This early recruitment likely contributed to the significantly stronger beta suppression apparent in this region already in the speech preparation phase. In other words, only AWS recruited the right mouth motor notably early in the speech preparation stage, while FS engaged the right mouth motor cortex closer to speech onset itself. A particularly intriguing and unexpected finding was that, compared to FS, AWS also had a significantly stronger increase of beta power in the same region of the mouth motor cortex bilaterally. Beta power increase appeared in the window preceding stimulus presentation and was significantly correlated between the left and right mouth motor cortices only in the AWS. Since no group differences were observed for alpha suppression of the auditory cortices, the discussion below will focus primarily on the differences observed in the beta range.

Suppression of beta power in the motor cortex has been observed to precede and accompany motor execution in a variety of self-paced speech and non-speech movement tasks (Bai et al., 2005; Doyle et al., 2005; Alegre et al., 2006; Erbil and Ungan, 2007; Tzagarakis et al., 2010). It is consequently widely acknowledged for its role in facilitating movement initiation (Pfurtscheller and Da Silva, 1999; Cheyne et al., 2006; Jenkinson and Brown, 2011). Recent studies have suggested that beta suppression preceding speech production reflects the formulation of a motor-articulatory plan and the feed-forwarding of this plan to sensory regions, which will in turn enable proper monitoring and feed-back during speech execution (Saarinen et al., 2006; Engel and Fries, 2010; Gehrig et al., 2012; Guenther and Vladusich, 2012; Hickok, 2012; Klimesch, 2012; Kilavik et al., 2013; Jenson et al., 2014). Critical to the current study is the consideration of factors that can modulate pre-movement suppression of beta power. Previous studies have demonstrated that pre-movement beta suppression can be increased by increasing task difficulty, either by demanding higher task speed, or by imposing a greater load opposing finger or hand extensions (Stančák et al., 1997; Pastötter et al., 2012; Nakayashiki et al., 2014). Increased motor task complexity also induced a stronger bilateral hemodynamic response in the motor cortex during unimanual movement, and particularly increased the recruitment of the ipsilateral motor cortex (Shibasaki et al., 1993; Catalan et al., 1998; Verstynen et al., 2005). An exaggerated bilateral hemodynamic response and beta suppression in the motor cortex has been observed in elderly participants completing motor tasks, findings that were interpreted to reflect facilitative mechanisms in a declining automaticity of the motor control network (Mattay et al., 2002; Wu and Hallett, 2005; Naccarato et al., 2006; Rossiter et al., 2014; Zimerman et al., 2014; Graziadio et al., 2015).

Based on these observations and supported by our current findings, we propose that exaggerated beta suppression preceding speech in our sample of AWS may be a reflection of reduced coordination in the motor network. Reduced automaticity in motor performance and motor learning has been widely implicated in the underlying mechanisms of speech-motor abilities of AWS (Prescott, 1988; van Lieshout et al., 1996; Van Lieshout et al., 2004; Smits-Bandstra et al., 2006; Smits-Bandstra and De Nil, 2007, 2009; Namasivayam and van Lieshout, 2008, 2011; Bauerly and De Nil, 2011), and over-activation of the motor cortex, particularly in the right hemisphere, has been frequently observed during speech execution in this population (Brown et al., 2005; Belyk et al., 2015). For this reason, the exaggerated motor engagement reported in our sample of AWS may not seem as particularly novel. However, the uniqueness of our finding is that group differences in beta suppression in the mouth motor cortex were observed following stimulus presentation and prior to actual speech onset, a time-window in which the feed-forwarding of the motor-articulatory plan is suggested to be taking place (Gehrig et al., 2012; Guenther and Vladusich, 2012; Hickok, 2012; Jenson et al., 2014; Liljeström et al., 2014). Our findings may therefore suggest that an impaired automaticity in the speech motor network, suggested by the current and past studies, may have consequences as early as in the speech preparation phase. Furthermore, although previous authors have suggested a functional role for the right motor cortex in stuttering due to its exaggerated activation during speech production (De Nil and Kroll, 2001; Preibisch et al., 2003; Chang et al., 2008), our study further found that engagement of this region in the AWS was significantly premature when compared to FS and began early in the speech preparation stage. This is particularly interesting as it is in this preparation stage when speech representations in the left inferior frontal gyrus are proposed to be translated to articulatory command codes in the left ventral premotor cortex (Papoutsi et al., 2009; Guenther and Vladusich, 2012; Hickok, 2012; Beal et al., 2015). In a recent study, Beal et al. (2015) found atypical gray matter maturation in the left inferior frontal gyrus (Broca’s area) in children and teens who stutter, and suggested that this coding of neural representations needed for formulating speech-motor commands is impaired in the stuttering population (Beal et al., 2015). Moreover, Chang et al. (2015) reported reduced and inconsistent development of white matter pathways interconnecting motor and auditory regions in children who stutter, particularly in the left primary motor cortex and medial temporal gyrus, as well as the left inferior frontal gyrus (Chang et al., 2015). The authors proposed that children who stutter experience reduced sensory-motor integration required to support speech production. It is therefore possible that the impaired translation between speech neural representations to articulation commands may be a contributing factor to the reduced speech-motor automaticity of AWS, which may have consequences on motor coordination in the speech preparation stage.

A further interesting finding in this study was the group difference in the increase of beta power, otherwise termed beta synchronization, which was observed before stimulus presentation in the same region of the bilateral mouth motor cortex. Beta synchronization has been associated with tonic muscle contractions, the successful withholding of a motor response, and with the slowing of voluntary movement execution (Gilbertson et al., 2005; Pfurtscheller et al., 2005; Engel and Fries, 2010; Jenkinson and Brown, 2011; Cheyne, 2013). It has also been frequently observed to occur at movement termination, following the beta suppression (Jurkiewicz et al., 2006; Alegre et al., 2008; Cheyne, 2013). Such findings have led to theories that beta synchronization may reflect an anti-kinetic component in the motor network that favors the maintenance of the current motor state and inhibits novel motor commands (Gilbertson et al., 2005; Engel and Fries, 2010). In our case the observed beta synchronization may be associated with speech movement termination as the participants completed their utterance and awaited the next stimulus. The strong positive correlation between the beta synchronization across the two hemispheres found only in the AWS suggests some functional role for this change. We hypothesize that stronger beta synchronization reflects a more strongly inhibited motor state baseline; the motor system returns to this inhibited state at the end of the speech act and consequently requires a greater effort to disengage from this baseline in order to facilitate speech production. This results in an exaggerated beta suppression when the next articulatory sequence is presented for speech preparation. There is some evidence to show that increasing motor task complexity exaggerates beta suppression during movement as well as post-movement beta synchronization (Stančák et al., 1997). It is therefore possible that the observed greater beta suppression and beta power increase in our group of AWS reflect the same effect of reduced speech-motor automaticity on the speech production task.

Our interpretation fits a computational model of stuttering where dysfluencies were proposed to be triggered by interruptions within a basal ganglia thalamo-cortical circuit responsible for syllable selection and motor planning (Civier et al., 2013). The model showed that dysfluencies can be caused either by failure to deactivate the previous selection, or by failure to activate the next motor plan in time. Indeed, anomalies in the internal timing networks have been recently proposed to underlie stuttering, suggesting that it may be associated with reduced ability to use predictive cues to control speech production (Alm, 2004; Etchell et al., 2014a,b; Wieland et al., 2015). If the AWS in our study were less able to predict the timing of the task, despite it being fairly rhythmic, their motor system may be less able to appropriately regulate itself to engage or disengage between the changing articulatory plans. In our data, this may have been reflected by the excessive beta synchronization. It is noteworthy that the FS barely showed any synchronization beyond the baseline. Instead, their beta time-course seemed to return to more of a flat line prior to the next stimulus presentation, particularly in the right hemisphere. In contrast, the motor system in the control subjects may have been better able to anticipate the upcoming stimulus and consequently did not fully “offset” at the end of every utterance, but was maintained in a more prepared state.

The lack of group differences in the engagement of the auditory cortex was surprising given the existing evidence of auditory-motor integration deficits in this population (Civier et al., 2010; Jansson-Verkasalo et al., 2014; Cai et al., 2014a; Tahaei et al., 2014; Daliri and Max, 2015). Yet despite the lack of a group difference, we observed that higher stuttering severity corresponded to reduced alpha suppression in the left auditory cortex, although rather weakly. Given the presence of only three severe participants in our sample of AWS and the weak relationship, it would be premature to draw any conclusions at this point. We would also like to point out that the predictability of the task timing, despite the 1–2 s jitter, may have affected the beta modulation time-course given the observed effect of consistent inter-stimulus timing on the slope of beta synchronization (Arnal, 2012; Fujioka et al., 2012). Fujioka et al. (2012) suggested that beta synchronization reflects the timely anticipation of an upcoming stimulus. However, any effect of rhythmicity should have modulated the beta response of both groups similarly, as they were subjected to the same inter-trial jitter. Lastly, with the large number of trials presented in this task, it is conceivable that some adaptation effect took place as the task progressed, particularly with regards to the timing of the task or with the degree of motor planning. Task adaptation could therefore affect motor preparation phenomena. Considering the evidence of slower motor learning in AWS (Namasivayam and van Lieshout, 2008, 2011), one hypothesis can be that earlier trials would show greater beta suppression in AWS with greater involvement of the right hemisphere. Controls, on the other hand, may not show as stark a contrast between early and late trials in such a relatively simple task. Our preliminary analysis of response latency times did not reveal any significant behavioral practice effects. Although it was not the focus of this current investigation, the effect of motor or other task adaptation can further inform of the role that oscillations play in the speech production of AWS.

This work highlights the importance of characterizing the speech-motor network assembly prior to speech onset in the stuttering population using high-temporal resolution brain imaging. As the current study only focused on perceptually fluent speech and excluded all stuttered trials, the findings are limited in their ability to explain contributing factors to a moment of dysfluency. We can hypothesize that exaggerated beta suppression is a strategic requirement to facilitate fluency in a motor system that is maladapted to do so consistently. If the beta synchronization observed before stimulus presentation indeed reflects a more strongly inhibited motor system, then it is possible that fluency is facilitated by overcoming this inhibited state with exaggerated beta suppression. Indeed, the role of hyperactive cortical beta oscillations has been proposed in one previous study on AWS during reading, where enhanced beta activity was notably reduced under delayed auditory feedback (Rastatter et al., 1998). In accordance with our hypothesis, we expect to see insufficient beta suppression preceding stuttered speech production when compared to fluent production. Similarly, comparing the unfolding of activation in the right motor cortex prior to dysfluent and fluent utterances would expand our understanding of the functional role of the right hemisphere in stuttering behavior.

## Author Contributions

A-MM: designed experimental protocol, carried out all data-collection, data-analysis, and write-up; approved and accountable for the presented manuscript. CJ: contributor to data-analysis, manuscript revision and approval of submission. DOC: author of brain imaging analysis software used in the current work and major contributor to data methodology, analysis, interpretation of results, manuscript revision and approval of submission. LDN: main contributor to conception and design of the work, data interpretation, manuscript revision, approval and accountability for the novelty presented in the work.

## Conflict of Interest Statement

The authors declare that the research was conducted in the absence of any commercial or financial relationships that could be construed as a potential conflict of interest.

## Footnotes

^1^http://www.psychopy.org/

^2^http://cheynelab.utoronto.ca/brainwave

^3^http://www.fil.ion.ucl.ac.uk/spm/software/spm8/

^4^www.talairach.org

## Abbreviations

AWS: Adults who stutter
ERD: Event-related desynchronization
ERS: Event-related synchronization
FS: Fluent speakers
MEG: Magnetoencephalography.
